# Use of Social Mobilization and Community Mobilizers by Non-governmental Health Organizations in Malawi to Support the Eradication of Polio, Improve Routine Immunization Coverage, and Control Measles and Neonatal Tetanus

**DOI:** 10.4269/ajtmh.19-0021

**Published:** 2019-10

**Authors:** Andrew Chimpololo, Vanessa Burrowes

**Affiliations:** 1 University of Malawi, Blantyre, Malawi;; 2 Johns Hopkins Bloomberg School of Public Health, Baltimore, Maryland

## Abstract

Seventy-five percent of children aged 12–23 months in Malawi have received all eight basic vaccinations—still leaving many children at risk. The Malawi Expanded Program on Immunization comprehensive Multi-Year Plan 2016–2020 reveals several challenges impeding immunization and disease surveillance efforts, such as the fact that non-governmental health organizations (NGHOs) and communities are minimally included in the planning, implementation, and monitoring of these activities. This article examines the extent to which NGHOs are promoting the use of social mobilization (SM) and community mobilizers (CMs) for sharing health information related to the eradication of polio, the importance of routine immunization, and the control of measles and neonatal tetanus. Data collection involved document analysis and interviews with 11 organizations in Malawi whose work contributes to the eradication of polio and control of measles and neonatal tetanus. Content analysis was used to analyze the qualitative data, whereas descriptive statistics were used to analyze the quantitative data. Non-governmental health organizations use a variety of approaches for SM, including mass media campaigns (radio and printed booklets), local skits and dramas, and home visits. Most NGHOs use training workshops and opinion leaders to impart knowledge and skills to CMs on immunization to eradicate polio and to control measles and neonatal tetanus. Major challenges faced by the NGHOs include negative attitudes toward campaigns and demotivation of CMs due to lack of financial incentives. The article concludes with a discussion of approaches to strengthen SM and the role of CMs by NGHOs.

## INTRODUCTION

The contribution of non-governmental health organizations (NGHOs) and their use of social mobilization (SM) and community mobilizers (CMs) are widespread but not well documented. We use the term CM in this article to refer to local volunteers working in communities under the supervision of NGHOs to promote health, including immunization-related activities.^1^ This article examines the extent to which NGHOs in Malawi use SM and CMs for sharing health information related to polio, measles, and neonatal tetanus prevention by increasing participation in immunization campaigns, improving routine immunization coverage, and detecting new cases of vaccine-preventable diseases. We use the term NGHO to include organizations that are not part of the government of Malawi but are engaged in SM and in the use of CMs. This includes traditional non-governmental organizations such as World Vision and Save the Children as well as other organizations such as the United Nations Children’s Fund (UNICEF), the United States Agency for International Development (USAID), and the University of North Carolina (UNC).

Although there has been significant improvement in vaccination coverage in Africa, challenges remain. For example, the goal of eliminating maternal and neonatal tetanus by 2015 was missed,^2^ and neonatal tetanus had only been eliminated in 34 of 47 (72%) of the WHO African Region Member States by 2015.^3^ The remaining 13 countries need additional support, and those that have achieved control need to sustain this achievement through the implementation of context-specific immunization and surveillance strategies. Mihigo et al.^4^ observed that 20–30% of the children in the African region remain either unimmunized or underimmunized because of supply-side and demand-side constraints.^4^ Social mobilization and CMs are underused resources in Africa for strengthening immunization programs in addition to other equally important interventions, such as ensuring that vaccination services are accessible, convenient, reliable, and friendly.

UNICEF defines SM as a broadscale movement to engage people’s participation in achieving a specific health goal through self-reliant efforts—those that depend on their own resources and strengths.^5^ Social mobilization is a holistic approach that involves all relevant segments of society: policymakers and other decision-makers, opinion leaders, the media, bureaucrats and technical experts, professional associations, religious groups, private sectors, NGHOs, community members, and individuals. It takes into account the collective needs of the people, embraces the critical principle of community involvement, and seeks to empower individuals and groups for action.^5^

UNICEF, the CORE Group Polio Project (CGPP), and their partners have used SM as the cornerstone of their strategy for polio eradication throughout the world.^6^ In recognition of the diverse interpretations globally of what SM entails, Obregon and Waisbord^7^ have identified three kinds of SM used in polio eradication efforts: 1) pragmatic SM, 2) activist SM, and 3) hybrid SM that combines both pragmatic and activist elements.

Pragmatic SM approaches use practical ways in which international health programs can engage community groups and leaders to pass along important information to intended beneficiaries and assist the program in performing other important tasks needed to achieve program goals. They involve using community members as instruments to help achieve predetermined goals, such as immunizing all children against polio or identifying cases of acute flaccid paralysis, measles, or neonatal tetanus.

Activist SM is characterized by community ownership and wrests the decision-making power from global or national direction to local communities that identify their own goals and strategies. Hybrid SM is a blend of pragmatic and activist elements that is characteristic of the most successful polio eradication efforts. Hybrid approaches combine the best aspects of both pragmatic SM (organizing and coordinating activities) and activist SM (harnessing leadership and insights within the community).^7^ Obregon and Waisbord^7^ further observe that combining them has resulted in greater success in reaching and immunizing children in high-risk populations. Murphy^5^ points out that in every country where the CGPP works, a blend of pragmatic and activist SM has been achieved. In addition to SM activities, the CGPP contributes funding and technical guidance to national and international collaborating groups—all aimed at strengthening host-country efforts to eradicate polio and improve routine immunization.

Malawi has made strong progress in expanding coverage of immunizations for childhood diseases. However, 25% of children in Malawi are still not fully vaccinated.^8^ Although Malawi attained polio-free status from the Africa Regional Certification Commission in 2005, performance of acute flaccid paralysis surveillance has not been meeting international standards.^9^ Cases of neonatal tetanus have also been documented in at least five districts since claims about its elimination were made in 2010.^10^

The most recent Malawi Demographic and Health Survey (carried out in 2015–16) indicates that 75% of children aged 12–23 months have received all eight basic vaccinations, which includes one dose each of Bacillus Calmette–Guérin (BCG) and measles, three doses of DPT/HepB/Hib (pentavalent vaccine against diphtheria, pertussis, tetanus, hepatitis B, and Haemophilus influenzae type b), and three doses of polio vaccine.^8^ Through a USAID program, the Malawi government used the Reaching Every Community approach to support immunization coverage in two low-performing districts (Dowa and Ntchisi), where the fully vaccinated valid dose coverage was much lower (60% in Dowa and 49% in Ntchisi).^11^ Malawi also experienced a major outbreak of measles in 2010, with 118,712 cases and 249 deaths. However, there have been no major outbreaks since 2010, and this success can be attributed to the implementation of catch-up campaigns.^9^

Most NGHOs in Malawi are adopting the SM approach for raising awareness on immunization. However, according to the 2015–16 Malawi Demographic and Health Survey,^8^ the drop in the immunization coverage level from 81% to 71% suggests the need for improvements in the overall immunization program, including the use of SM and CMs.

The use of CMs is critical to achieve success in programs that apply hybrid SM as they provide a link between NGHOs and the community. The use of CMs in surveillance and public awareness campaigns in Malawi has successfully worked in other health-related areas. For instance, family planning policy and program implementation involved the training of health surveillance assistants (HSAs) and volunteer community-based distribution agents (CBDAs) in the provision of family planning services and specific contraceptives at the community level, where the number of clients accessing injectable contraceptives rose from 3,210 in 2009 to 101,885 in 2011.^12^ The HSAs are salaried Ministry of Health (MOH) personnel, whereas the CBDAs are CMs who work under the supervision of HSAs. The HSAs and CBDAs counsel clients on family planning methods, and they promote the utilization of services when a mobile family planning team visits the community.^10^

## METHODS

A mixed-methods approach using quantitative and qualitative methods of data collection and analysis was adopted for investigating the use of CMs by NGHOs in immunization programs in Malawi.^13,14^ We carried out internet searches and consulted with the Malawi MOH to identify NGHOs implementing programs for the eradication of polio, promotion of immunizations, and control of measles and neonatal tetanus. The process identified a total of 11 NGHOs, all of which participated in this study: UNICEF, the Catholic Relief Services (CRS), the Catholic Development Commission in Malawi (CADECOM), the Catholic Health Commission, World Vision International (WVI) in Malawi, CARE International, UNC, the Adventist Development Relief Agency (Malawi), Save the Children, Amref Health Africa (Malawi), and USAID in Malawi.

### Data collection.

Data collection for this article consisted of data extraction from documents, questionnaire surveys, and semistructured interviews. Documents were collected from the MOH’s database and from an internet search of the 11 NGHOs included in the study. A questionnaire was administered to the field staff members from the 11 organizations listed previously. The interviews explored the extent to which the organizations were using CMs in their programs and the approaches that the CMs were using to convey health information and promote immunization campaign participation as well as routine immunization. Each of the 11 participating organizations completed the questionnaire. Questions focused on the public health successes achieved by the NGHOs through their use of CMs and SM, challenges they faced, and suggested solutions. The survey also included questions regarding the extent to which the organizations were using tools, strategies, and innovations promoted by the CGPP.

Semistructured interviews were also conducted between November 2017 and January 2018 with senior staff of four NGHOs–Care International, Save the Children, CADECOM, and the Catholic Health Commission–to obtain further insights into the use of CMs. One of us (A. C.) interviewed one senior officer from each of these four organizations. We had originally planned to interview senior officers from all the 11 organizations, but representatives from seven organizations were not available. Semistructured interviews were carried out to complement the findings of the structured questionnaire by raising spontaneous questions and obtaining more detailed information than was possible using a structured questionnaire.^15^ The questions for these semi-structured interviews focused on the extent to which NGHOs in Malawi adapted the best practices from other countries to the local context, the level of planning within the NGHOs, and coordination among NGHOs.

### Analysis.

We carried out a cross-methods analysis to compare the results across the three data collection methods.^16^ In addition, quantitative measures were identified from the data extraction process, although the research was predominantly qualitative.^17^ Content analysis was used to analyze the qualitative data, whereas descriptive statistics were used to analyze the quantitative data. To determine which SM approach the organizations were uisng, we decided that an organization was applying pragmatic SM approaches if its programs involved the use of community groups or community leaders to convey information to the target audiences. We decided that organizations were applying activist SM, in addition to other approaches, if they engaged local communities in identifying their own goals and strategies in the programs for ownership purposes in the planning and coordination stages, and finally, those using hybrid SM demonstrated that they combined pragmatic and activist SM activities in their programs.

### Ethical considerations.

Before the research, the authors obtained ethical approval from the University of Malawi (Polytechnic) Research Ethics Committee. All study participants were provided with an informed consent process before completing the questionnaire survey and before participating in the semistructured interview.

## RESULTS

### Social mobilization strategies for use of CMs adopted by NGHOs.

Our findings from this study indicate that UNICEF/Malawi, the Catholic Health Commission, Save the Children, WVI/Malawi, and CADECOM integrate different SM techniques into their programs for both routine immunization and SM campaigns. UNICEF/Malawi focuses on interpersonal communication provided by HSAs, mass media communications, and “edutainment” on Child Health Days. “Edutainment” is a community mobilization technique that combines education and entertainment activities. It is used to educate the community on health and immunization. Immunizations and vitamin A supplementation are provided as well. The 2016 CGPP Annual Report provides further evidence that SM techniques were transferred from other countries to Malawi.^18^ UNICEF’s implementing partners, MaiKhanda and PACHI, carry out SM activities in the communities to orient family members on sexual/reproductive health, immunization, and other health issues.^19^ Family members support HSAs in identifying pregnant women and linking them with services.

The Catholic Health Commission conducts SM activities through HSAs who train CMs on antenatal care. Catholic Development Commission in Malawi uses drama, songs, poetry and dance, as well as other community participatory approaches to convey health information.

Save the Children organizes open days (open-air events organized in a community to share information with everyone), at which time SM is implemented for immunization and family planning activities. The primary target audience is women who bring their children to clinics where measles and polio immunizations are provided. World Vision/Malawi relies mostly on household-level counseling through Care Group Volunteers (CGVs), who are basically local volunteers in the communities, to relay health messages.^20,21^ They also use community-level meetings, open days, and media campaigns.

The CRS consortium also uses the Care Group model for SM, which makes it possible to communicate health information to every household through a cascade process of information sharing. An officer from CARE International described this process as follows:

“The model involves training facilitators, known as Care Group Promoters, who orient lead parents selected within the communities to act as peer educators for 10-12 neighbors. The lead parents are volunteers who train their fellow community members and conduct public awareness on immunization through community meetings and house-to-house visits.”

### Descriptions of SM techniques and CM strategies.

USAID/Malawi fully adopts the CGPP model in its projects. A national team of master trainers facilitates capacity-building programs for district community mobilization trainers and the district health promotion subcommittee of the District Executive Committee. These people subsequently train community mobilization teams. Community mobilization also involves the use of media campaigns transmitted through radio programs and printed booklets to pass health information encouraging immunization and antenatal visits.^22^ The community mobilization teams also link community groups to radio programs through radio listener clubs. These teams conduct public outreach activities to encourage access to health services, including immunizations. Adventist Development Relief Agency/Malawi focuses on health and nutritional improvement for children and uses facilitators to train community members and implement advocacy activities as part of its SM approach. Amref Health Africa conducts its SM through public meetings and training workshops to raise awareness about maternal and newborn mortality.

### Deployment of SM and CMs in high-risk areas.

Two districts, Dowa and Ntchisi, have had persistently low immunization coverage. The USAID-funded Maternal and Child Survival Program targets these two districts.^22^ Whereas the national Expanded Program of Immunization in Malawi targets all 29 districts in the country equally, the CRS UBALE project operates only in Blantyre Rural, Chikwawa, and Nsanje districts. All these programs use CMs in passing health information to the respective communities.

### Extent of usage of SM and CMs.

The questionnaire results reveal that 91% (10 out 11) of the organizations use training workshops, usually for health field staff, on how CMs can impart knowledge and skills on immunization to eradicate polio as well as to control measles and neonatal tetanus. Fifty-four percent of the organizations in our study make extensive use of CMs to share health information, 27% make moderate use of them, and 19% do not use them at all. Seventy-two percent of the participating organizations have their own communication strategies, which include health interventions concerning immunizations. UNICEF and USAID do not have their own communication strategies but support the MOH’s communications strategy, which includes interventions on immunizations.

The results show that the participating organizations use various strategies in their SM activities: 17% of the NGHOs use the hybrid SM approach, 52% use the pragmatic SM approach, whereas 31% use the activist SM approach. In terms of specific techniques, 64% use house-to-house visits, 82% engage influencers (opinion leaders), and 55% engage women as CMs. Eighty-four percent of the organizations track women and children to register pregnancies and births as well as to identify unimmunized children, 64% of the organizations also involve community groups in their campaigns, and 9% work with schools and students.

Community meetings, open days, and media campaigns reach large audiences within a short period of time and promote community action. The use of drama enables community members to contextualize messages and convey them in the local language. Radio listening clubs, which are community groups established to listen to radio shows and discuss them, are an effective form of communication that links mass communication with interpersonal communication. Printed materials complement communication by mass media and interpersonal means.

### Challenges affecting SM and the use of CMs.

Occasionally, household members have negative attitudes toward campaigns or interpersonal conflicts with CMs. These can affect the receptivity of households to visits from CMs. Another challenge is that men are less likely to take part in training sessions for CMs and to participate in the open days. Exposure of communities to varying campaign approaches and methodologies from different organizations also leads to confusion. In addition, not only is retention of radio messages low but radio listening clubs are also difficult to scale up because of logistical and financial challenges. Another major challenge affecting immunization campaign success is demotivation of CMs because of lack of financial incentives. In some cases, CMs expect financial benefits and quickly lose motivation if this expectation is not met. One of the people interviewed stated the following:

“The CMs always complain that the per diem given to them is insufficient for their upkeep. This affects their performance and some of them quit midway before their work is completed.”

## DISCUSSION

This study highlights various techniques of SM among 11 NGHOs in Malawi for SM and for the use of CMs. These NGHOs are using a variety of SM techniques to share health information, including those intended to improve immunization coverage rates. Most organizations use CMs to mobilize members of the community to take part in immunization activities. The meetings conducted by the CMs provide a platform for clarifying misconceptions related to immunizations.

Similar activities take place in other countries. In India, for example, the CGPP hires block mobilization coordinators to train community mobilization coordinators to provide SM for polio eradication.^5^ (See also other articles in this series about the CGPP in India.^23–25^) The community mobilization coordinators are similar to Care Group Promoters in the Care Group model because their main responsibility is to train CMs. These block mobilization coordinators are trained in interpersonal communication skills to help them express sincere friendliness and helpfulness to skeptical or suspicious caregivers while conveying accurate and reassuring information.^5^ The training workshops involve talks, role plays, and guidelines that enable the community mobilization coordinators to learn about the importance of asking about the caregivers’ health and well-being and taking time to listen to them.

Although the programs of some of the organizations included in this study do not directly focus on the eradication of polio and control of measles and neonatal tetanus, their activities do embrace community-based surveillance which targets infectious diseases such as polio and measles. The integration of community-based surveillance for infectious diseases, other medical conditions, and vital events registration (of births and deaths) has supported polio eradication efforts in India,^25^ Ethiopia,^26^ and South Sudan.^27^

Our findings suggest a need to institute a subcommittee of the Inter-agency Coordinating Committee for the control of vaccine-preventable diseases in Malawi. The membership of this committee should include all organizations carrying out SM through CMs. According to the national Expanded Program on Immunization, NGHOs have contributed significantly to the improvement of child health in Malawi by supporting the provision of all recommended immunizations to 460,000 infants in 2016.^19^ In addition, the HSAs were able to provide essential health services and conduct mass screening of 1.9 million children in 2016 using SM techniques.^19^ The coverage for the diphtheria–pertussis–tetanus (DPT) vaccine also showed improvement, with 84% of children vaccinated with DPT against a baseline of 81% and a target of 93%. World Vision/Malawi has also trained 15,940 CGVs and more than 150,000 households were reached by CGVs in 2015.^28^ Through USAID’s Maternal and Child Survival Program, the use of SM has increased the utilization of immunization and family planning services in more than 90% of health facilities in the priority districts of Dowa and Ntchisi.^22^ In addition, more than 180 facility staffs, 100 Area Development Committee members, and 500 community members have received orientations on immunization and family planning integration.

### Contribution of civil society organizations, NGHOs, and communities to basic health services in Malawi and other African countries.

Participating organizations gain much from the use of CMs for sharing health information. The NGHOs claim that CMs are easily accepted by the communities because they are from the community and people feel freer to interact with them than with formally trained health-care workers, who are often not from the local village. However, further studies are needed to confirm this. During interviews, officers from the participating organizations stated that when the communities are intricately involved in the implementation of health campaigns, they take ownership of the process, help contextualize campaign activities, and help to relate the programs to the needs of their local communities.

The use of community meetings and house-to-house visits by the CRS consortium members who use the Care Group model provides evidence of the transfer of the CGPP innovations to Malawi from other countries. In India, the CGPP has initiated house-to-house visits to educate caregivers of unimmunized children about the importance of receiving oral polio vaccine and of engaging them to fully immunize their children.^5,25^ In addition, such visits are the first strategy used because some caregivers simply need information about when and where vaccination booths for special campaigns will be set up or how to access routine immunization services.

Some organizations such as UNICEF and CADECOM also use drama for development activities and “edutainment” to disseminate health information. Theater performances help the NGHOs attract large crowds where messages can be communicated in understandable and memorable ways using the local language, and they stimulate discussions that motivate people to bring their children for vaccination. Furthermore, people may discuss barriers to immunization and identify ways to address them. Repetition of important health information from varying sources is effective in promoting behavioral change. The utilization of drama as a strategy for communicating health information indicates that some NGHOs are using best practices from other countries and applying them to Malawi. More needs to be done to encourage organizations to incorporate such activities into their programming.

Immunization and family planning integration during open days primarily targets women who bring their children to special under-five clinics for measles and polio immunization. Just as in India and Nigeria, public awareness activities are included in the open day programs to attract people to the clinic. Open days are usually complemented by media campaigns, which involve running radio and television advertisements and sharing special information, education, and communication materials. These materials, which are distributed during SM events, contain messages about routine immunizations. Although these information, education, and communication activities have been helpful in increasing access to routine immunizations in India, they, by themselves, do not appear to be sufficient to achieve high levels of routine immunization coverage in Uttar Pradesh.^29^ Longer term relationships of trust between caretakers and local health workers (including CMs) are required as well.^29^ As in the Uttar Pradesh case, strategies for promotion of immunization in the predominantly Muslim eastern region of Malawi need to be tailored to dissipate misconceptions about vaccinations.

The findings from our study indicate that each SM intervention comes with its own challenges. Health education talks in group settings provide limited opportunities for audience feedback, making it difficult to determine whether participants retain the information being conveyed. However, such talks (as well as drama) can be an excellent opportunity for audience feedback, group reflection on the feasibility of carrying out the recommendations, discussions of barriers and ways to overcome them, and commitments from individuals to try to carry out recommended actions. The development of appropriate messages for theater communication can be difficult because of the nuances of the local vernacular from one place to another. Mass media campaigns are expensive and cannot reach remote and/or mobile populations that lack access to radios or televisions. Interpersonal communication through health workers can result in fatigue from high workloads. Brochures and handouts are expensive to produce and ineffective for illiterate community members. “Edutainment” requires substantial resources to organize, making it costly for the implementing organization.

It became apparent during the interviews that unsalaried, temporary positions for CMs often produce discord in the SM processes. Despite getting clear communication that the position is unsalaried, some CMs still expect financial support. When their demands are not met, the CMs either slow down or quit altogether. Community mobilizers often receive no payment beyond a modest per diem, which is similar to practices in other CGPP countries.^30^ The morale among CM Coordinators in India, on the other hand, is generally high in large part because they are full-time paid workers.^5^ In the Horn of Africa, CMs are unsalaried, but they receive nonmonetary incentives such as uniforms or bags for supplies.^27^ In some contexts such as Angola, CMs receive financial incentives for maintaining records of children in their catchment areas and encouraging household caregivers to have their children immunized.

The NGHOs included in our study do not target mobile, remote, or refugee populations. Refugees from Mozambique, Burundi, and South Sudan reside in camps in Malawi where immunization programs are managed by relief organizations. The CGPP’s extensive work in India demonstrates that SM can contribute to high-quality infectious disease surveillance and immunization for underserved and at-risk poulations.^5^ In Malawi, the NGHOs could benefit from the involvement of schools and students in their immunization activities to extend the reach of immunization work. The CGPP experience in Angola, Ethiopia, and India indicates that when mothers are absent, older children become the caretakers of younger siblings, so peer education became a mobilization strategy.^5^

Our findings highlight the value that a subcommittee on vaccine-preventable diseases would bring the control of vaccine-preventable diseases in Malawi by more fully engaging civil society and all NGHOs carrying out SM and using CMs. Such a committee could potentially strengthen SM and CM activities, avoid duplication of efforts, strengthen funding mechanisms, and fortify monitoring and evaluation mechanisms.

## CONCLUSION

Social mobilization and CMs are widely used in Malawi, although in a somewhat fragmented manner. Non-governmental health organizations have used SM and CMs to contribute to the eradication of polio, promote immunizations, control measles and neonatal tetanus, and address other health priorities. The major SM strategies used by NGHOs in Malawi include open community meetings, health education talks, household visits, brochures and other handouts, mass media, and theater for development. Our research indicates that the enhancement of SM and the use of CMs could help address the recent decline in immunization coverage in Malawi.

Our review indicates that NGHOs are transferring some of the best practices of the CGPP from other countries to Malawian context. However, more could be done to use women as mobilizers, engage teachers and students, and include mobile and remote communities as target populations in immunization campaigns. This study has contributed knowledge on the extent to which NGHOs in Malawi are using SM and CMs as recommended by the CGPP for polio eradication and for the control of vaccine-preventable diseases. The organizations carrying out SM and using CMs might consider establishing a subcommittee on vaccine-preventable diseases, thereby strengthening SM and CM activities.
